# 

**Published:** 1901-09-07

**Authors:** 


### NOTICE TO CORRESPONDENTS.

All MSS., Letters, Books for Review, and other matters intended for the Editor should be addressed The Editor, "The Hospital," The Hospital Building, 28 & 29 Southampton Street, Strand, W.C.

The Editor cannot undertake to return rejected MS., even when accompanied by stamped directed envelope.

**Notice to Advertisers and Subscribers.**

All Advertisements, Orders for Copies of The Hospital, and Business Communications should be addressed to **THE MANAGER (Not to the Editor),** The Hospital, 28 & 29 Southampton Street, Strand, London, W.C.

The Cost of Subscription, post free, is as follows:—Per week, 2½d.; per quarter, 2s. 3d.; per half-year, 6s. 3d.; per annum, 10s. 6d. Foreign:—Per annum, 15s. 2d., payable, in all cases, in advance.

**NOTICE.—Vol. XXIX. of "The Hospital" (October 1900, to March, 1901), in handsome cloth binding is now on sale at the Office of this Journal, price, 7s. 6d. Cases for binding the Numbers contained in this Volume 1s. 6d. Index to Vol. XXIX., 2½d., post free.**

**The Monthly Part for September is now ready. Price 6d., by post 8d.**

### The Student of Medicine.

### APPOINTMENTS.

L. Eminson, L.F.P.S.Glasg., L.S.A. has been appointed District Medical Officer of Cosford Union. H. Hine has been appointed District Medical Officer of Guildford Union. N. Hay Forbes, F.R.C.S.Edin., J.P., has been appointed Honorary Consulting Surgeon to the Black Rock Convalescent Hospital and Consulting Surgeon to the Rother Valley Railway Company. C. F. Laing, M.B., C.M.Glas., has been appointed Medical Superintendent to the Somerset and Bath Asylum. H. P. Miles, M.R.C.S., L.R.C.P.Lond., has been appointed District Medical Officer of Kingsbridge Union.

MEDICAL HISTORY FROM THE EARLIEST TIMES.

By E. T. WITHINGTON, M.A., M.B. Oxon.

Demy 8vo., over 400 pp., with two Plates, cloth gilt, 12/6 net.

"One of the best attempts that has yet been made in the English language to present the reader with a concise epitome of the history of medicine, and as such we very cordially commend it."

*Glasgow Medical Journal.*

THE SCIENTIFIC PRESS, LTD.,
28 & 29 Southampton Street, Strand, London, W.C.

